# Comprehensive profile and contrastive analysis of circular RNA expression in cervical squamous carcinoma and adenocarcinoma

**DOI:** 10.7717/peerj.14759

**Published:** 2023-01-26

**Authors:** Hongxue Luo, Yi Zhu, Jiaqi Wang, Yue Wang, Lihui Wei

**Affiliations:** Department of Gynecology and Obstetrics, Peking University People’Hospital, Peking University, Beijing, China

**Keywords:** Circular RNA, Expression profile, Cervical squamous carcinoma, Cervical adenocarcinoma, High-throughput RNA sequencing, Tumor differentiation, Bioinformatics analysis, Ubiquitin mediated proteolysis

## Abstract

**Background:**

Numerous studies have shown circular RNA (circRNA) dysregulation is associated with the pathogenesis of cervical cancer,particularly in individual carcinoma variants. The aim of this study is to investigate and contrastively analyze the expression pattern of circRNAs in cervical squamous carcinoma and adenocarcinoma mediated by human papillomavirus type 16 (HPV-16).

**Methods:**

The expression of circRNAs in cervical squamous carcinoma (SCC), adenocarcinoma (ADC) and adenosquamous carcinoma (ASC) tissues, together with the adjacent normal tissues (ANT), was profiled by high-throughput RNA sequencing (RNA-seq). Bioinformatics analysis and quantitative real time polymerase chain reaction (qRT-PCR) validation of the sequencing data were performed. A network of circRNA-miRNA (microRNA)-mRNA was then constructed according to predicted targets and function of candidate circRNAs.

**Results:**

A total of 11,685 annotated circRNAs were identified in six cervical samples. There were 42 up-regulated and 98 down-regulated circRNAs. 215 circRNAs were up-regulated in SCC but down-regulated circRNAs in ADC, while 50 circRNAs displayed the opposite trend. Function enrichment analysis based on different expressions of circRNAs found that the most enriched pathway in all the three pathologic variants of cervical cancer was the “ubiquitin mediated proteolysis” pathway. Eight key candidate circRNAs derived from this pathway were further validated, and we noticed that several target miRNAs of candidate circRNAs could target the source genes. Based on this we constructed a related competing endogenous RNA (ceRNA) network.

**Conclusion:**

Through a comprehensive interpretation of differentially expressed circRNAs in different pathologic variants of cervical cancer, this study provides new insights into the process of tumor differentiation mediated by HPV. Our results may help to complement the molecular typing and stem cell theory of cervical cancer.

## Introduction

Cervical squamous cell carcinoma (SCC) and cervical adenocarcinoma (ADC) are two major histological variants of cervical malignant tumors (Cree et al., 2020). Almost all SCC and over 85% of ADC are associated with human papilloma virus (HPV) infections, particularly HPV type 16 and 18 (De Sanjose et al., 2010; Pirog et al., 2000). In the past decades, HPV vaccination programs and screening strategies of primary HPV have dramatically reduced SCCs worldwide (Bray et al., 2018; Schiffman et al., 2011). Yet, ADC appears to be increasing in both absolute and relative incidence, especially among young women (Bray et al., 2005; Chen et al., 2016; Siegel, Miller & Jemal, 2020). While it was previously thought that the two variants had completely different pathogenesis, an accumulating body of research indicates that about 50% of high-grade squamous intraepithelial lesions (CIN), a precancerous lesions of the cervix, merged cervical adenoepitheliopathies (Miller et al., 2015; Park et al., 2011), and early-stage SCC tissues contain residual CIN and adenocarcinoma *in situ* of cervix (AIS) (Lee & Flynn, 2000). The bidirectional transformation of cervical malignant stem cells is currently being uncovered (Colgan & Lickrish, 1990; Smedts, Ramaekers & Hopman, 2010; Yao et al., 2015). Yet it is still unclear in which variant a cervical tumor would evolve into, nor what signal molecules dominate this process and the mechanisms that are behind it.

Several studies have used high-throughput genotyping platforms to address the integrated genomic and molecular characterization of SCC and ADC, shedding light into genes mutation more frequently present in SCC, such as PIK3CA, and others, such as KRAS mutations, that are almost uniquely restricted to ADC patients (Ojesina et al., 2014; Wright et al., 2013). Researchers have used oligo-microarray and pathway analysis to describe the transcriptomic signature and molecular networks associated with SCC and ADC, demonstrating that some genes (KRT17, IGFBP2, *etc*.) are differentially expressed in ADC and SCC (Oh et al., 2012). A study based on DNA microarray analysis has identified more differentially expressed genes (12-LOX, TRY2, *etc*.) between ADC and SCC (Contag et al., 2004), and found a number of genes (CEACAM5, TACSTD1, *etc*.) only expressed in ADC (Chao et al., 2006). It is hard to assess whether these genes and variations are the cause of the differentiation and progression of cervical cancer, or if they are caused by it. We aim to investigate this process by focusing on circRNA, a class of endogenous noncoding RNA, through the profiling of the characteristic and expression of circRNAs in SCC and ADC.

CircRNAs are conserved and abundantly expressed in various eukaryotic cells. They have covalent bonds between the 3′ head and the 5′ tail ends that cause the RNA to close into a loop that is resistant to RNA exonuclease and is structurally stable (Kristensen et al., 2019; Memczak et al., 2013; Rybak-Wolf et al., 2015). CircRNAs can act as sponge molecules using their miRNA response elements (MREs) to bind to miRNAs in order to regulate the expression of downstream target mRNAs, and interact with RNA-binding protein (RBP) to form RNA protein complex (RPC), which regulates the transcription of linear parent genes (Holdt, Kohlmaier & Teupser, 2018). In addition, circRNAs can bind ribosomes to form a complex that directly regulates gene transcription and participates in protein translation (Greene et al., 2017).

Emerging evidence suggests circRNAs are involved in the development and progression of multiple cancers (Hou & Zhang, 2017; Kristensen et al., 2022), including cervical cancer (Chaichian et al., 2020). To these days, several studies have been profiling circRNAs in SCC (Huang et al., 2020; Wang et al., 2017; Xu et al., 2020b) and ADC (Xu & Lu, 2021; Xu et al., 2020a) using high-throughput RNA sequencing (RNA-seq) and bioinformatics analyses. These descriptive studies have demonstrated differentially expressed circRNAs between cervical cancerous tissues and normal tissues may play key roles in the tumorigenic process. To the best of our knowledge, this is the first comparative analysis study on the circRNAs profiles of SCC and ADC, and adenosquamous carcinoma (ASC). In our research, we aim to employ RNA-seq data to investigate similarities and differences of circRNA expression profiles between SCC and ADC, and to identify regulatory circRNAs behind CC potentially involved in tumorigenesis and differentiation.

## Materials & Methods

### Specimens preparation

Three pairs of samples of stage IB1 cervical cancer tissue and adjacent normal tissue (ANT) were selected for RNA-seq from three HPV-16 positive patients with SCC, ADC or ASC (all negative to other HPVs). ADC was limited to endometrioid adenocarcinoma, the usual type of adenocarcinoma, and ASC was limited to the term with both squamous and glandular areas that were each clearly recognizable without the use of special stains, the usual type of adenosquamous to carcinomas, according to histopathology. Diagnosis was based on the International Federation of Gynecology and Obstetrics (FIGO) criteria. For surgery samples, tumor tissues were taken from the center of the tumor. The corresponding adjacent noncancerous cervical tissues were taken at least two cm away from the edge of the cancer. All tissues were placed in liquid nitrogen immediately after being dissected and stored the same way. Other six pairs of samples of HPV-16 mediated SCC and adjacent normal tissues, and six matched HPV-16 mediated ADC samples, were used for quantitative real time polymerase chain reaction (qRT-PCR) validation. All procedures were approved by the ethics committee of the Peking University People’s Hospital (NO.2019PHB212-01), and written informed consent was obtained from all patients.

### RNA extraction and quality control

Total RNA was extracted using TRIzol reagent (Invitrogen, Carlsbad, CA, USA) following the manufacturer’s protocol. The integrity of RNA was evaluated by standard denaturing agarose gel electrophoresis and using the Agilent 2100 bioanalyzer (Agilent Technologies, Palo Alto, CA, USA). Purity and concentration, as well as the preliminary quantification, were determined using a Nano Drop spectrophotometer (Nano Drop, Wilmington, DE, USA).

### Library building for RNA-seq

After removing ribosomal RNA and building a special library, RNA-seq data collection was conducted using the Illumina PE150 platform by Novogene Bioinformatics Technology Co. Ltd. (Beijing, China). Clean reads for subsequent analyses were obtained through raw data filtering, sequencing error rate check and GC content distribution check. Clean reads were then compared and mapped to the reference genome. Find_circ and CIRI2 were used to detect and identify circRNAs (Gao et al., 2017; Memczak et al., 2013). Following identification of circRNAs, length distribution and sources of known or novel circRNAs were counted. Density statistics and circRNA locations on each chromosome were identified by Circos software for all circRNAs of each sample, and compared to all chromosomes (Krzywinski et al., 2009). Normalization of readcount by TPM was conducted before expression analysis (Zhou et al., 2010).

Raw sequencing data has been uploaded to NCBI’s Gene Expression Omnibus (GEO) and is accessible through GEO Series accession number GSE208089.

### Bioinformatic analysis

Differentially expressed circRNAs between three paired samples (S_1 *vs.* S_2 (cervical squamous cell carcinomas tissue *vs.* paired adjacent normal tissue), A_1 *vs.* A_2 (cervical adenocarcinoma tissue *vs.* paired adjacent normal tissue), AS_1 *vs.* AS_2 (adenosquamous carcinoma of cervix *vs.* the paired adjacent normal tissue)) were determined by negative binomial distribution test using the DESeq R package (Anders & Huber, 2010), with criteria: |log2(fold change)| > 1 and *q* value < 0.05. Three other extrinsic matched combinations were explored CCT_S *vs.* ANT_S (cervical cancer containing squamous cell carcinoma tissues *vs.* corresponding adjacent normal tissues, S_1&AS_1 *vs.* S_2&AS_2), CCT_A *vs.* ANT_A (cervical cancer containing adenocarcinoma tissues *vs.* corresponding adjacent normal tissues, A_1&AS_1 *vs.* A_2&AS_2), CCT *vs.* ANT (cervical cancer containing three pathological types above *vs.* corresponding adjacent normal tissues,S_1&AS_1&A_1 *vs.* S_2&AS_2&A_2), with *p* adj < 0.05. *P* value < 0.05 was only adopted when the differentially expressed circRNA were too little.

Based on the threshold values, we performed a hierarchical clustering analysis and generated a series of volcano plots in order to filter circRNAs with modified expression levels. The IRESfinder software based on the logit model was used as a prediction tool to determine the coding potential of circRNAs (Zhao et al., 2018). Subsequently, we predicted the interactions between circRNAs and miRNAs using the miRanda databases, and the interactions between miRNAs and genes of the eight candidate circRNAs using TargetScan v7.2 (Agarwal et al., 2015; Enright et al., 2003).

Gene Ontology (GO) analysis *via* GOseq software was carried out (Young et al., 2010). Kyoto Encyclopedia of Genes and Genomes (KEGG) pathway analysis was performed with the Kobas3.0 software to detect the involvement of circRNAs genes in different biological pathways (Kanehisa et al., 2008; Mao et al., 2005). We used the DIANA-miRPath v3.0 and the DIANA-microT-CDS algorithm to determine the functions of target genes involved in different biological pathways (Paraskevopoulou et al., 2013; Vlachos et al., 2015). The significance threshold was set at *p* value < 0.05 and FDR value < 0.01. The network connecting candidate circRNAs, target miRNAs and the mRNAs of host genes was generated and displayed *via* Cytoscape v3.6.1 software (https://cytoscape.org/).

### Quantitative real-time PCR

To validate circRNAs data generated from RNA-seq, ten significantly dysregulated circRNAs in all six comparisons (|log2(fold change)| > 2) were randomly selected for qRT-PCR. Other eight candidate circRNAs extracted from functional enrichment analysis were also selected. We designed divergent primers across the circular junctions as listed in Table S1. Total RNA was reverse transcribed using the Invitrogen Superscript cDNA Synthesis kit (Invitrogen, Carlsbad, CA, USA). CircRNA expression was measured through qPCR (SYBR Green PCR Master Mix; Applied Biosystems, Foster City, CA, USA). Reactions were performed in triplicate according to the manufacturer’s protocol. Relative circRNAs expression levels were calculated *via* the 2^−ΔΔ*Ct*^ method and *β*-actin was used as the housekeeping gene. Results are expressed as mean ±  SD (Standard Deviation). Statistical analysis was conducted using the SPSS Statistics 19.0 software. Significant difference between comparison was defined as a minimum 2-fold change in normalized expressed level with *p* value < 0.05.

## Results

### General expression profiles of circRNAs in different types of cervical cancer

**Figure 1:**
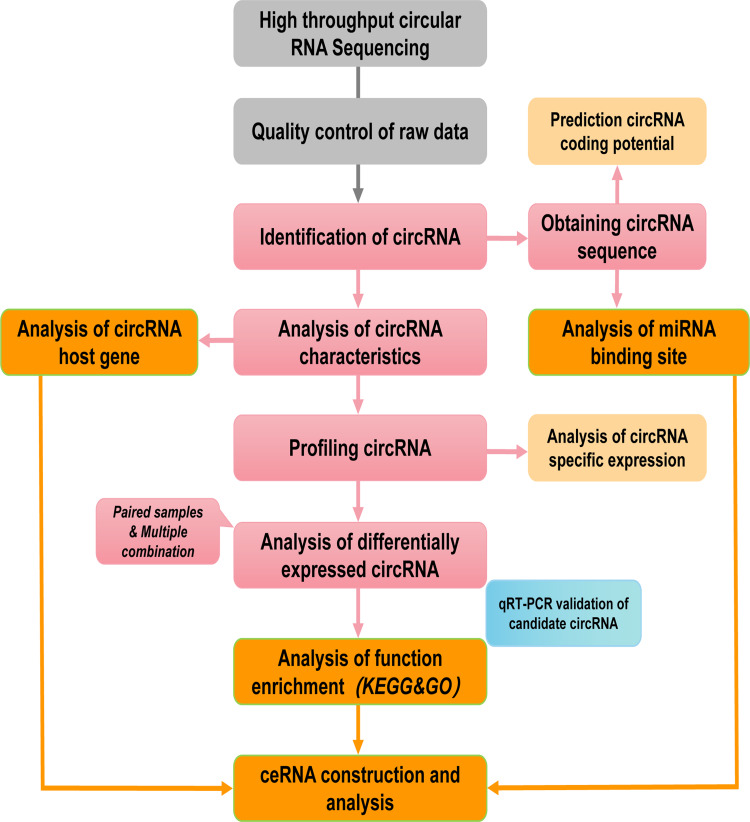
Pipeline for circRNAs sequencing and analyzing. The grey and pink boxs indicated the main steps of sequencing and the orange boxes contained main biological functional analysis of sequenced data.

**Figure 2:**
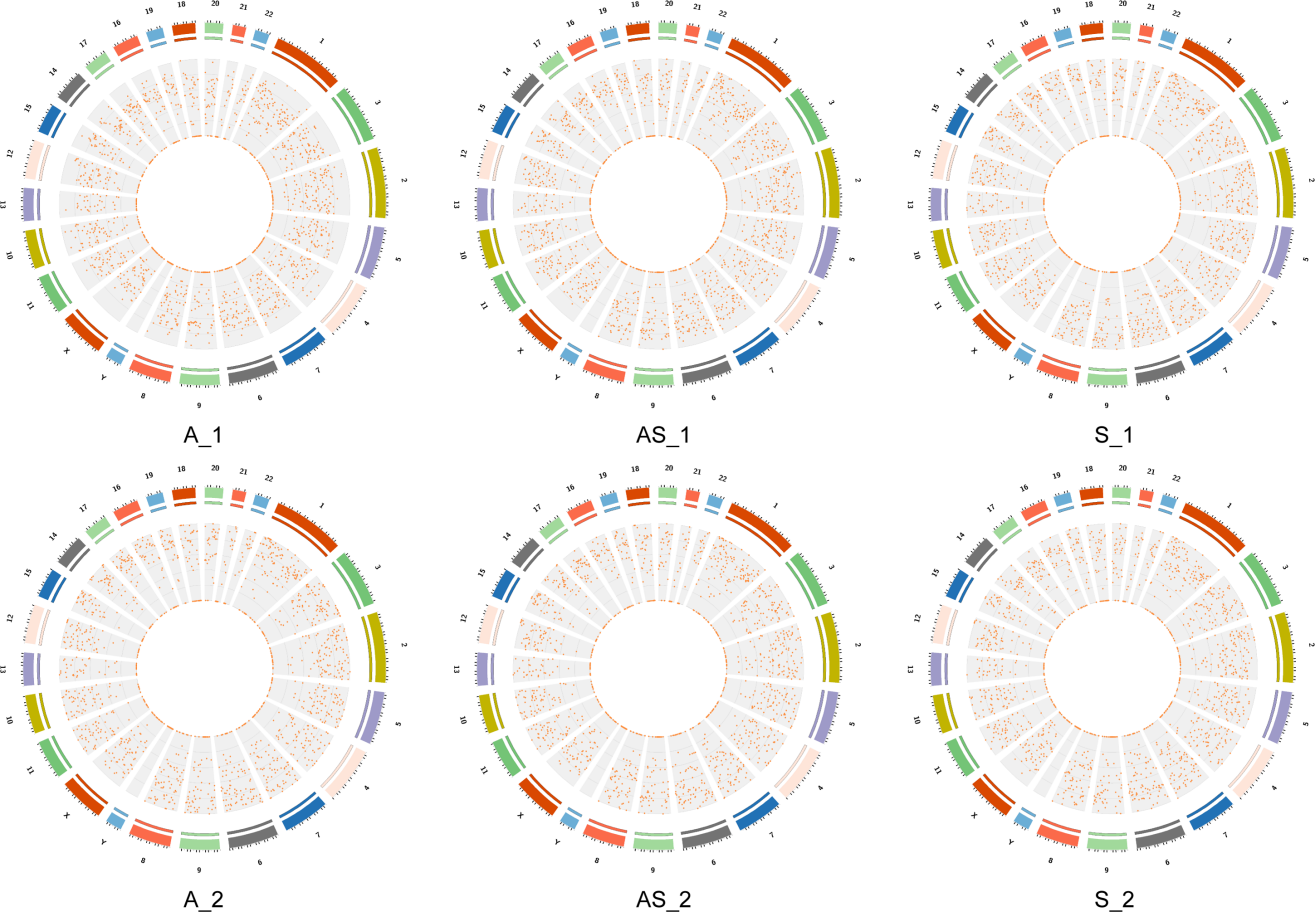
Locations of circRNAs identified on human chromosomes. The outer circle represents chromosome location with numbers outside. Different colors indicate different chromosomes, and the scale corresponds to coordinates, unit = M (millions). The inner circle represents interval circRNA density by a scatter diagram, where each point represents a coordinate interval in the chromosome. The value diminish from the outside in.

Identity and abundance of circRNAs of three paired samples of cervical cancer and adjacent normal tissues as described in the Methods section were investigated by RNA-seq, as shown in Fig. 1. From sequencing we identified a total of 27,148 circRNAs, 11,685 of which had annotations in the circBASE database. Based on genomic features and locations, we observed that the lengths of spliced circRNAs were mostly below 1200nt, and the corresponding genes mainly consisted of exonic and intronic sequences, while a small subset was derived from intergenic sequences. CircRNAs were distributed across all autosomes, as well as chromosome X, as shown in circos plots (Fig. 2). We found Chr 1,2,3 contained more circRNAs than the others. A full list of the identified circRNAs is available in File S1, including annotations, chromosomal locations, strand orientations, and source genes. In view of the accumulating evidence suggesting circRNAs have coding potential, data was analyzed and the predicted results of the identified circRNAs are shown in File S2.

**Figure 3:**
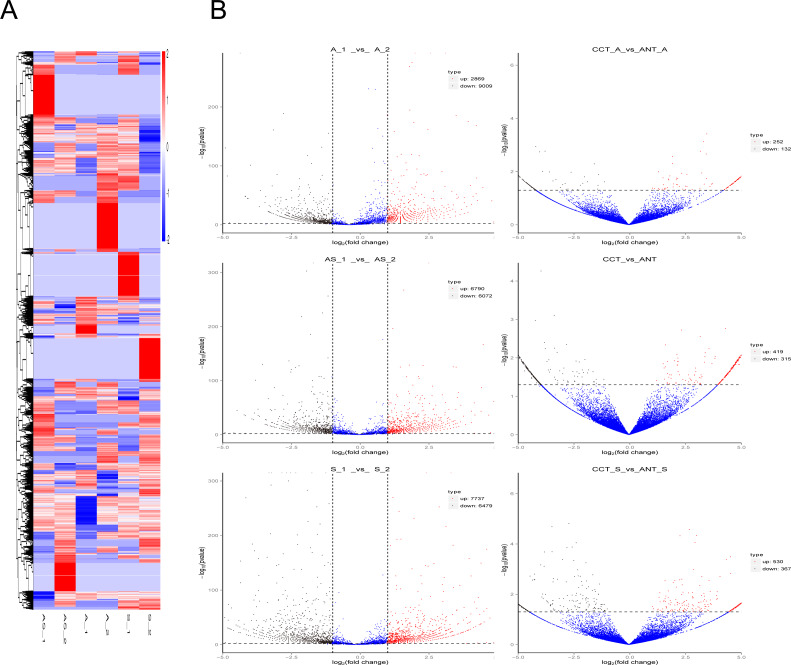
Differentially expressed circRNAs in the three comparison groups. (A) Heatmap of circRNAs patterns and cervical samples (rows and columns, respectively) on the basis of the normalized expression value according to the color key. High and low expressions are marked in red and blue. The color ranges from red to blue, indicating log10 (TPM + 1) from large to small. (B) Volcano plots of circRNAs expression. The two vertical lines represent the differential expression of 2-fold up and down, while the horizontal coordinate line represents *p* = 0.05. Black and red dots indicate low and high expression with statistical difference, and blue dots represent expressions with no significant difference.

Differential circRNA clustering analysis was used to determine the clustering pattern of differentially expressed circRNAs in different comparisons. We used the TPM value of differential circRNA sets in each sample for hierarchical clustering analysis, and for K-means and SOM clustering analysis (File S3). Discrimination among different comparison groups is shown in the cluster heatmap (Fig. 3A).

Volcano plots were created to visualize differentially expressed circRNAs between the six comparison groups (Fig. 3B). When we raised the threshold to 4 fold-change, we identified 42 up-regulated and 98 down-regulated circRNAs in the three intrinsic paired samples (A_1 *vs.* A_2, AS_1 *vs.* AS_2, and S_1 *vs.* S_2). If statistics was done in combination with the other three groups of comparison (CCT_A *vs.* ANT_A, CCT_S *vs.* ANT_S, CCT *vs.* ANT), there were only 10 and eight circRNAs left for further analysis, respectively (Table 1).

**Table 1:** Biological information of the top 10 upregulated or downregulated circRNAs.

**CircRNA_ID**	**Chr location**	**Length**	**Source gene**	**log2.Fold_change**						*P* value^**^
				**S_1 *vs.* S_2**	**AS_1 *vs.* AS_2**	**A_1 *vs.* A_2**	**CCT_S *vs.* ANT_S**	**CCT_A *vs.* ANT_A**	**CCT *vs.* ANT**	
up_expressed group^*^										
hsa_circ_0070648	4	342	SEC24B	6.112	4.766	6.475	4.474	4.407	5.364	0.004
hsa_circ_0027966	12	1703	SLC41A2	6.626	7.704	6.212	6.340	6.108	6.474	0.000
hsa_circ_0077817	6	272	PTPRK	4.626	6.640	5.475	4.857	4.929	5.235	0.005
hsa_circ_0037710	16	218	DNAJA3	6.392	4.181	7.212	4.557	4.989	5.757	0.002
hsa_circ_0023555	11	303	C2CD3	6.005	7.181	6.475	5.740	5.787	6.111	0.001
hsa_circ_0003861	9	319	CEMIP2	5.305	7.351	2.975	5.655	3.474	3.587	0.006
hsa_circ_0057510	2	972	NAB1	6.305	7.268	7.060	5.917	6.126	6.406	0.000
hsa_circ_0039052	16	290	ITGAL	7.059	7.088	6.698	6.163	5.822	6.458	0.000
hsa_circ_0001356	3	292	SMC4	5.112	6.640	4.890	5.004	4.728	5.206	0.006
hsa_circ_0042952	17	340	UTP6	6.112	6.181	4.890	5.131	4.282	5.300	0.004
down_expressed group^*^										
hsa_circ_0025969	12	622	SCAF11	−3.161	−6.887	−5.208	−3.496	−4.223	−3.515	0.020
hsa_circ_0002115	19	256	ZNF528	−4.348	−7.209	−6.101	−4.245	−4.756	−4.197	0.001
hsa_circ_0073128	5	871	HOMER1	−3.313	−7.025	−6.860	−3.516	−4.948	−3.784	0.004
hsa_circ_0054226	2	713	SLC8A1	−2.192	−3.689	−6.308	−2.349	−3.273	−2.332	0.023
hsa_circ_0003690	5	1103	SMAD5	−5.880	−7.266	−5.723	−5.631	−4.681	−5.454	0.003
hsa_circ_0005816	X	603	N/A	−4.465	−6.653	−5.723	−4.683	−4.202	−4.812	0.011
hsa_circ_0002815	1	2087	ATP2B4	−6.202	−6.472	−6.793	−5.238	−4.600	−5.511	0.002
hsa_circ_0003844	12	466	PLEKHA5	−6.340	−6.373	−6.208	−5.259	−4.224	−5.328	0.004

**Notes.**

* |log2(fold change)| > 2.

** The *p* of other five groups all arrived <0.05, here shown *p* of the group (CCT *vs.* ANT).

N/A not available

There were 215 circRNAs up-regulated in SCC (S_1 *vs.* S_2), but down-regulated in ADC (A_1 *vs.* A_2), and 50 circRNAs down-regulated in SCC (S_1 *vs.* S_2), but up-regulated in ADC (A_1 *vs.* A_2),. All circRNAs were equally expressed (|log2(fold change)| < 1) in ADC and in the corresponding ANT (File S4).

### Confirmation of results with RNA-seq for differentially expressed circRNAs

**Figure 4:**
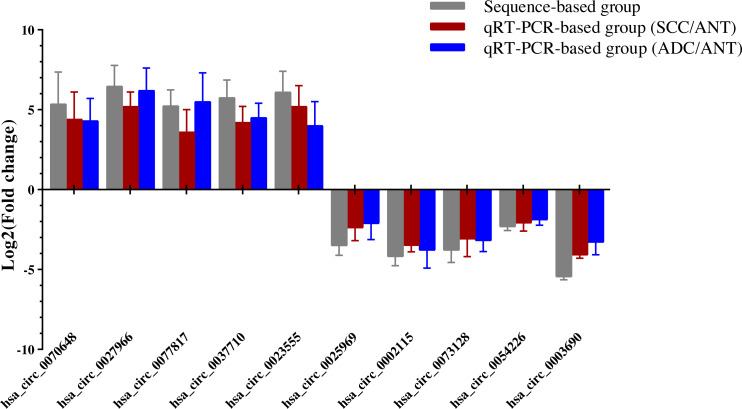
QRT-PCR conformation of differentially expressed circRNAs of RNA-seq. qRT-PCR was used to validate the dysregulated expression of the ten significantly up-regulated or down-regulated circRNAs in six pairs of HPV-16 mediated SCC & ADC and their adjacent normal tissues. Data is displayed as mean ± SD.

Ten overlapping significantly expressed circRNAs were randomly selected to confirm the reliability of our sequencing results: five up-regulated and five down-regulated level > 4 fold circRNAs in the six comparisons described above (Table 1). We validated the expression levels in six SCC and ADC samples *versus* their corresponding ANT samples. The results revealed that qRT-PCR-based fold-changes mirrored those observed in the sequencing data (Fig. 4, File S5).

### Comprehensive analysis of function enrichment derived from different comparisons

**Figure 5:**
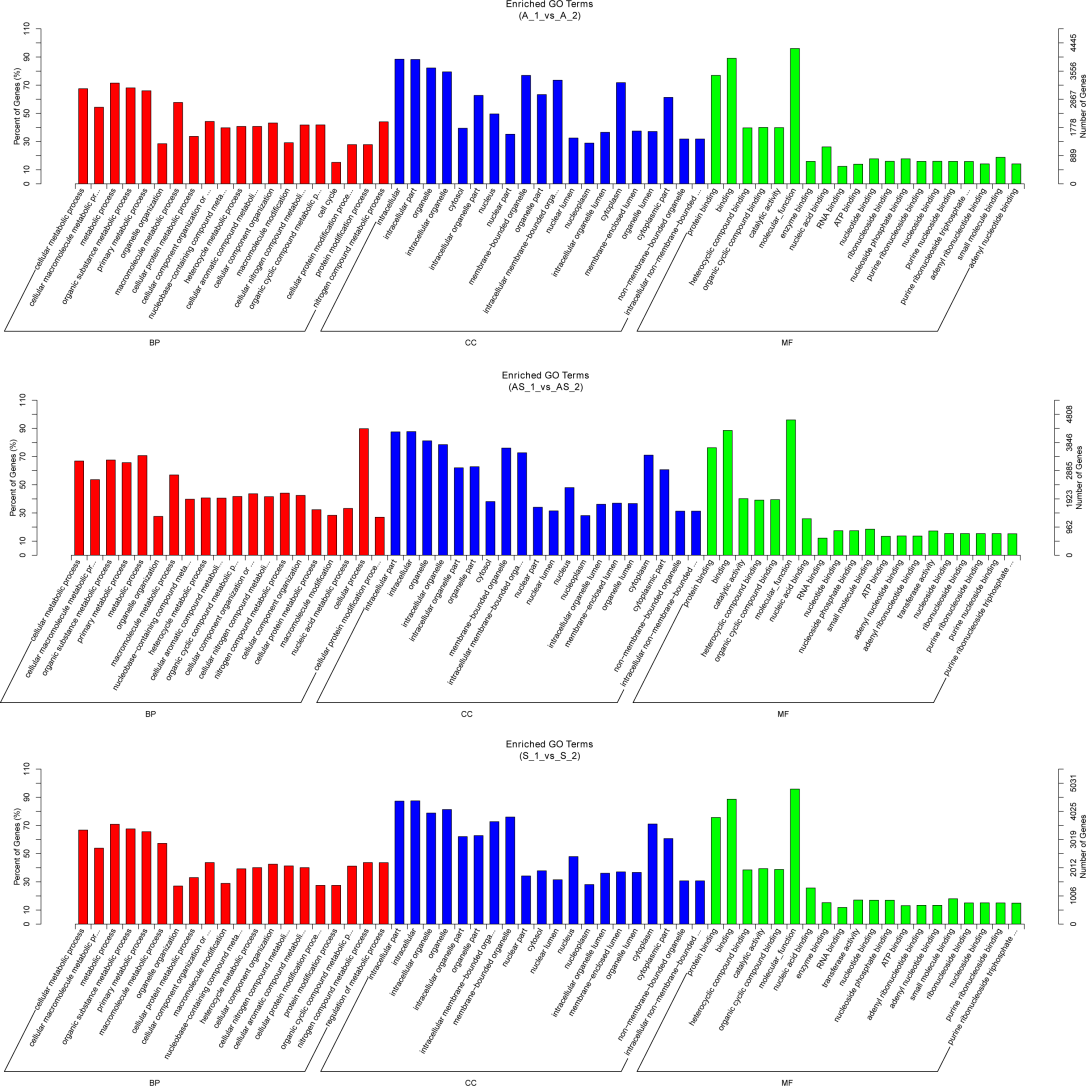
Gene ontology enrichment analysis of differentially expressed circRNA gene symbols. Top 20 GO terms are shown in three categories. BP, biological process; CP, cellular component; MF, molecular function.

**Figure 6:**
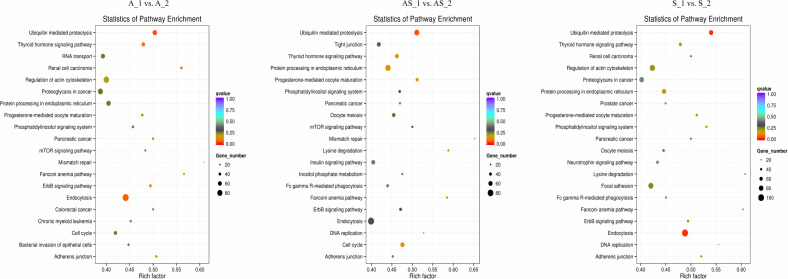
KEGG enrichment analysis of differentially expressed circRNA gene symbols. Top 20 signaling pathways involved in cervical tumorigenesis. The size of each dot indicates the number of circRNAs. The color of the dot indicated the variable range of *q* value.

In order to predict potential biological functions of differentially expressed circRNAs, we conducted GO and KEGG enrichment analysis of the relative genes (“source genes” or “host genes”) in three intrinsic comparison groups. We found the most significant terms in all GO categories (BP/CC/MF) were consistent among pathologic variants of cervical cancer, except the BP term “organelle organization” in ASC (Fig. 5). KEGG pathway analysis was conducted to determine the involvement of host genes in different biological pathways. We found the most enriched pathway in all the three pathologic variants of cervical cancer was “ubiquitin mediated proteolysis” (Fig. 6). In addition, KEGG analyses were performed on the reversely expressed circRNAs in SCC and ADC as listed in File S4, details are shown in File S6.

### Relative expression level detection of key candidate circRNAs

**Figure 7:**
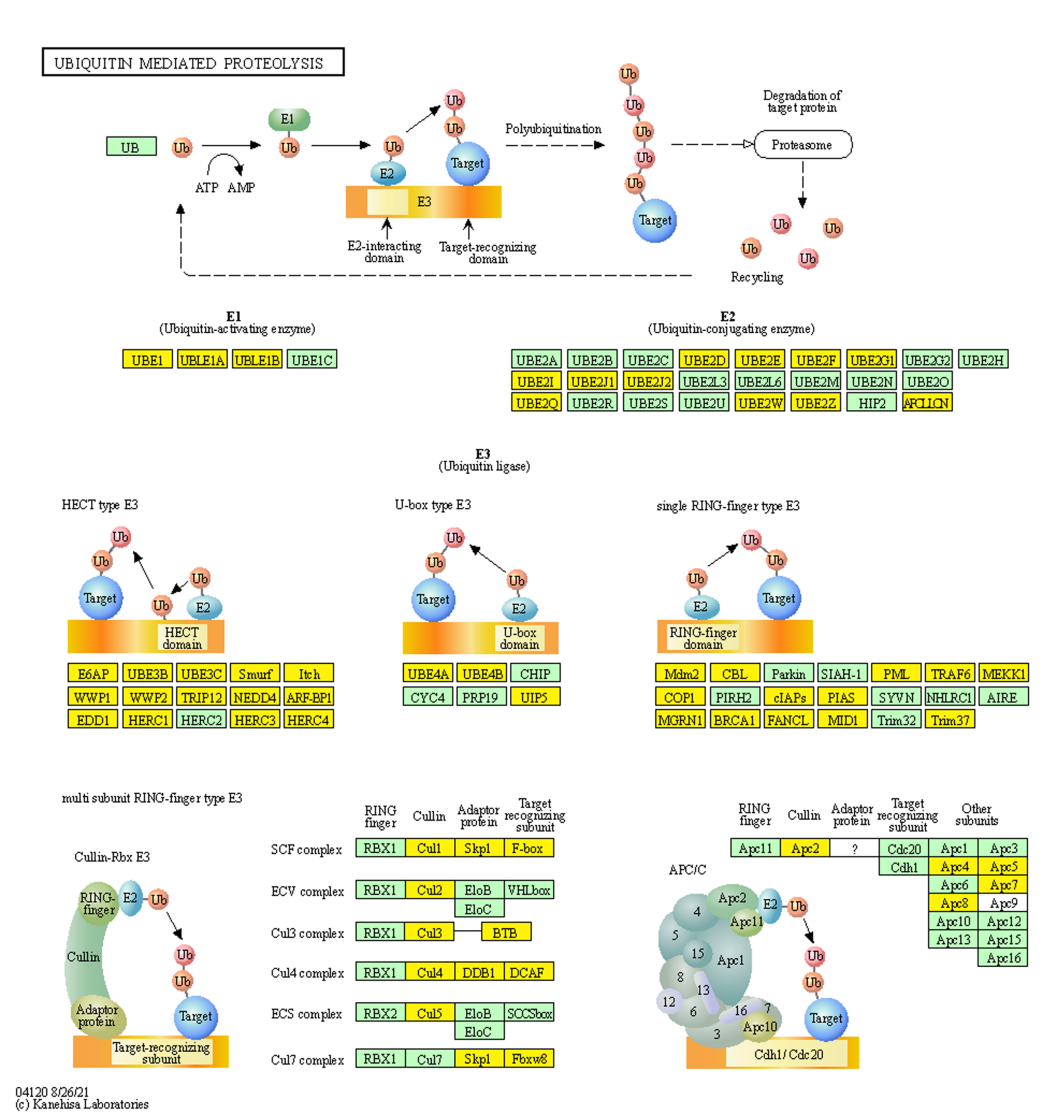
Genes associated with cervical cancer in the ubiquitin mediated proteolysis signaling pathway. Common genes involved are highlighted in yellow. The map was generated based on the DAVID Functional Annotation Tool and KEGG pathways.

In the three intrinsic comparisons considered, we found there were 69, 70, and 74 genes being involved in the “ubiquitin mediated proteolysis” pathway, 54 of which were common among all three (Fig. 7, File S7). Among these 54 circRNAs, we considered eight circRNAs that had a significant common dysregulation trend in the three intrinsic comparisons, or they had completely reverse expression trend between SCC and ADC but were equally-expressed (|log2(fold change)| < 1) in ASC (Table 2).

**Figure 8:**
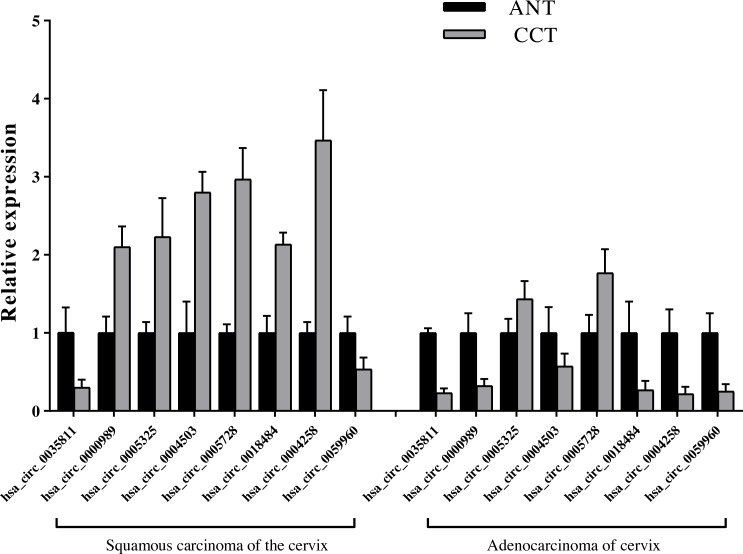
Identification of relative expression level of key candidate circRNAs with qRT-PCR. Expression level of cervical cancer tissue (CCT) was normalized to adjacent normal tissue (ANT). *n* = 6 per group. Data are displayed as mean ± SEM.

We detected the eight key candidate circRNAs by qRT-PCR, and identified that hsa_circ_0005325 and hsa_circ_0005728 were up-regulated in SCC and ADC compared with the ANT, hsa_circ_0035811 and hsa_circ_0059960 were down-regulated in SCC and ADC compared with the ANT. In addition, we found that the relative expression levels of the four circRNAs, hsa_circ_0000989, hsa_circ_0004503, hsa_circ_0018484, and hsa_circ_0004258, were up-regulated in SCC, but down-regulated in ADC compared to ANT, which is consistent to our sequencing results (Fig. 8, File S5).

### Construction of a ceRNA network based on candidate circRNAs and their host genes

**Figure 9:**
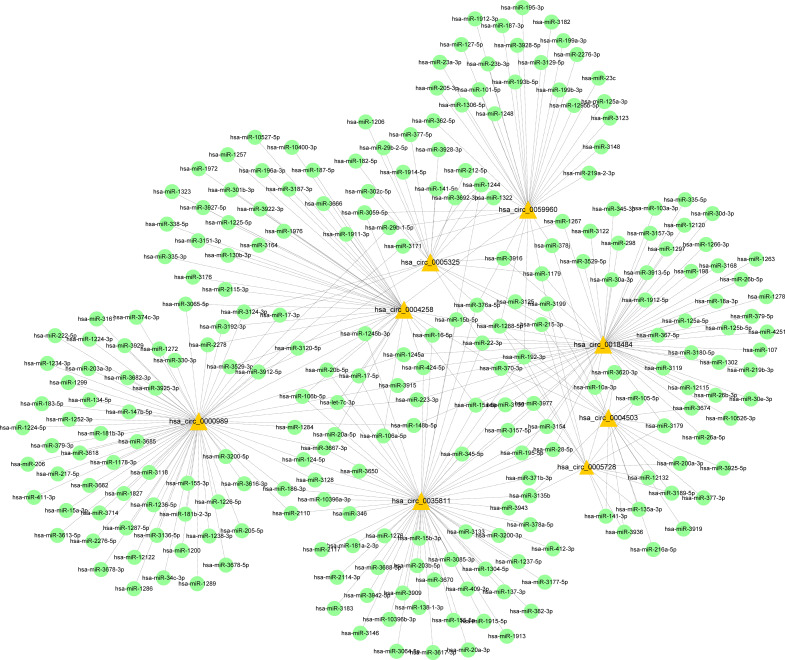
CircRNA–miRNA-host gene network in cervical cancer based on ubiquitin mediated proteolysis. Connectivity between eight candidate circRNAs and target miRNAs.

**Figure 10:**
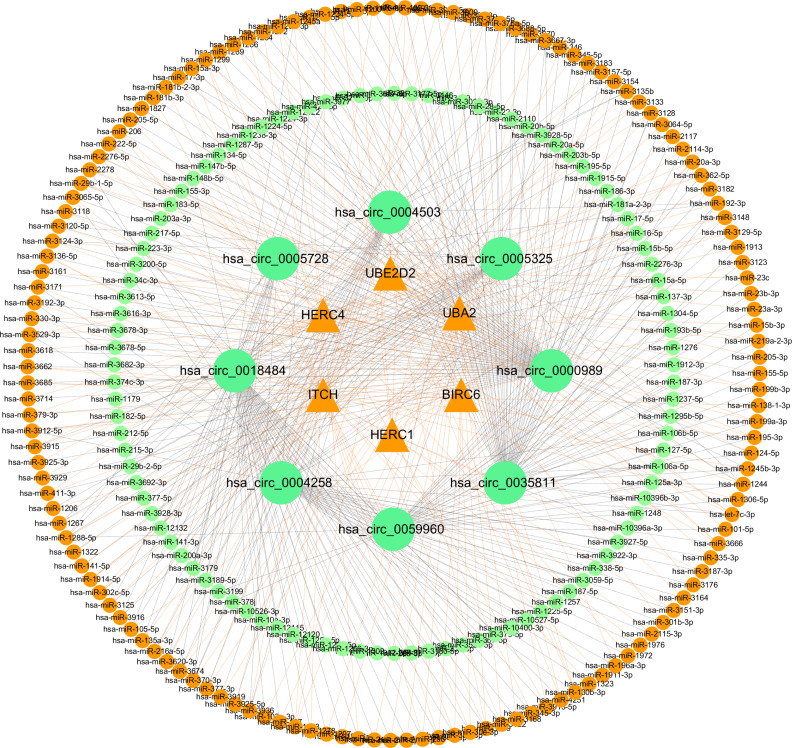
Network of ceRNA among host genes. The network consists of 245 miRNAs, eight circRNAs and their host genes.

**Figure 11:**
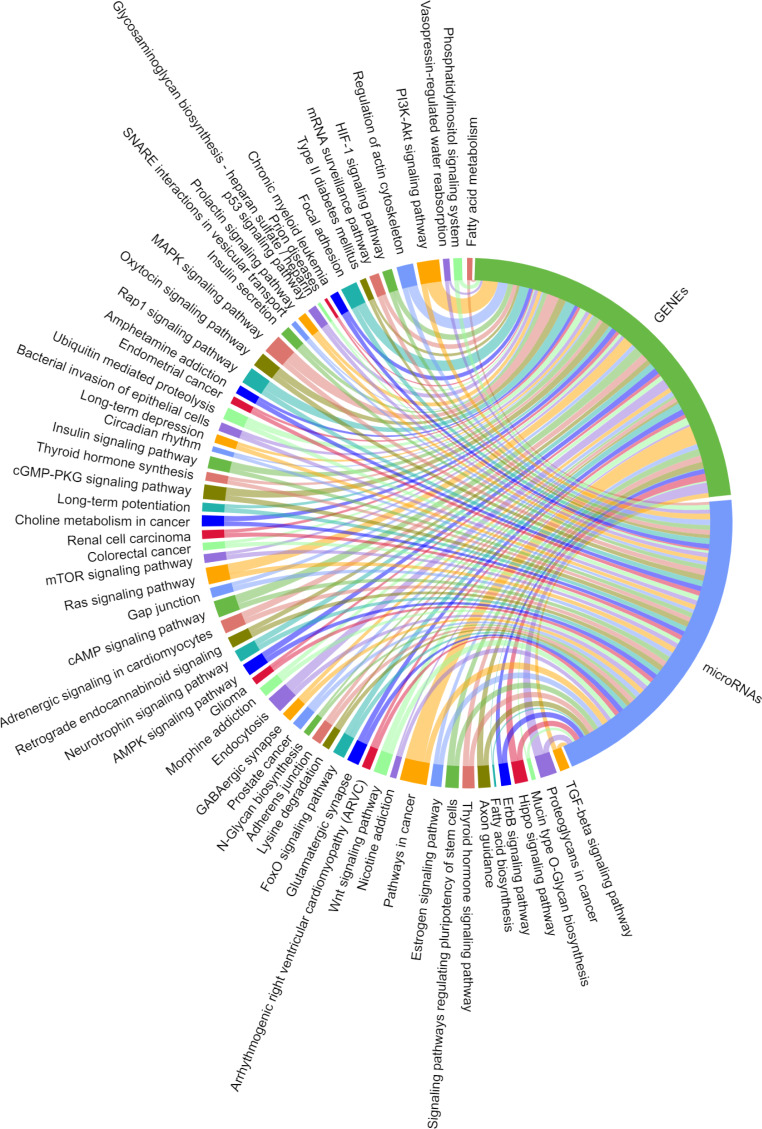
Significantly enriched KEGG pathways for the target mRNAs of miRNAs irrelevant to ubiquitin mediated proteolysis. A total of 102 miRNAs targeted by the eight candidate circRNAs, KEGG analysis was performed on their target mRNAs. Significantly enriched KEGG pathways feature *p* values < 0.05. Each line represents a gene (miRNA or mRNA), while the number of lines indicates the genes enriched.

We predicted the target miRNAs of the eight key candidate circRNAs (File S8), and built a target pool containing 245 miRNAs using the miRanda databases. A total of 143/245 miRNAs were overlapped with the target miRNAs predicted by TargetScan v7.2. Two interaction networks were constructed to reflect the relationships of ceRNA with Cytoscape v3.6.1 (Figs. 9 & 10). With a particular focus on the 102 miRNAs that showed no relation with the “ubiquitin mediated proteolysis” biological pathway, we generated a visual chordal graph to demonstrate the functional enrichment of miRNA and target genes using the Novomagic online platform tool (https://magic.novogene.com) (Fig. 11). TGF-beta signaling pathway, the most significant pathway, involved 61/102 miRNAs and 66 target genes. The profile data derived from KEGG pathway analysis by DIANA-miRPath v3.0 and with DIANA-microT-CDS algorithm are shown in File S9.

## Discussion

CircRNAs are single-stranded RNA transcripts characterized by covalently closed loop structures. They are mostly generated from pre-mRNAs through the process of “backsplicing” and have distinct properties compared to functional RNAs and other ncRNAs, such as the lack of the N7-methylated guanosine capping structure at 5′ end and polyadenylated tail at the 3′ end (Rahmati et al., 2021; Taheri et al., 2021; Wilusz, 2018). circRNAs are classified into four subtypes based on the genome region they originate from: exonic circRNAs (ecircRNA), intronic circRNA (ciRNA), exonic-intronic circRNA (EIciRNA), and intergenic circRNAs (Meng et al., 2017). The existence of circRNAs was first reported in 1979 following the microscopic identification of circular forms of RNA extracted from the cytoplasm of HeLa cervical tumor cells (Hsu & Coca-Prados, 1979). The recent rapid development of bioinformatics and RNA-seq led to the identification of thousands of circRNAs in mammals cells and tissues, and to the determination of their expression information in different tissues and developmental stages (Greene et al., 2017). A large body of evidence has demonstrated differentially expressed circRNAs are usually associated with the occurrence and progression of in various cancers, suggesting they have prognostic, diagnostic and therapeutic potentials (Najafi, 2022).

**Table 2:** Biological information of the eight candidate circRNAs.

**Circbase ID**	**Chromosome**	**Length (bp)**	**Source gene**	**log2.Fold_change**				**Expression level**
				**S_1 *vs.* S_2**	**AS_1 *vs.* AS_2**	**A_1 *vs.* A_2**	**CCT *vs.* ANT**	**S/AS/A**
hsa_circ_0035811	15	1052	HERC1	−4.8802	−4.8874	−5.8601	−4.2235	Down/down/down
hsa_circ_0000989	2	1152	BIRC6	1.3238	−0.043703	−8.686	−0.074847	Up/no/down
hsa_circ_0005325	19	321	UBA2	1.7911	2.282	0.21646	1.6299	Up/up/up
hsa_circ_0004503	5	374	UBE2D2	3.4174	−0.19918	−1.1294	0.7176	Up/no/down
hsa_circ_0005728	5	280	UBE2D2	2.7107	2.6597	0.63603	1.5751	Up/up/up
hsa_circ_0018484	10	898	HERC4	1.0093	−0.8795	−8.2833	−0.752	Up/no/down
hsa_circ_0004258	10	551	HERC4	5.8895	0.61526	−5.4006	0.63404	Up/no/down
hsa_circ_0059960	20	524	ITCH	−1.1607	−5.1505	−5.9856	−2.4809	Down/down/down

In our study, we used RNA-seq to systematically analyze circRNA profiles in SCC, ADC, and ASC. Our aim was to explore which circRNA are expressed concurrently or alternatively in different histological variants of cervical cancer. It is currently accepted that the persistent infection by oncogenic HPV of the cervical epithelium is the major cause of cervical cancer (Okunade, 2020; Reich et al., 2017). The tumor’s trend of developing into squamous or glandular may depend on the multipotential differentiation characteristics of cervical reserve cells under the columnar epithelium (Bekkers et al., 2003; Colgan & Lickrish, 1990; Park et al., 2000; Yao et al., 2015). This hypothesis is based mainly on morphologic considerations, yet definitive evidence about the mechanisms involved in this process is scarce. This study provides novel insights from the perspective of circRNAs variant-specific expression profiles.

Three pairs of variants of cervical cancer tissues and their adjacent normal tissues were selected for RNA-seq. In order to identify differentially expressed circRNAs, we considered six groups of comparisons, three intrinsic paired groups and three extrinsic matched combination groups. We found 18 abnormally expressed circRNAs (>4 fold change) concurrently occurring in all six comparison groups, of which 10 were up-regulated and eight down-regulated. The results about expression were highly consistent between qRT-PCR and RNA-seq. We identified circRNAs reversely dysregulated in SCC and ADC compared with ANT, but equally expressed (|log2(fold change)| < 1) in ASC *vs.* ANT. We hypothesize that the equal expression is the result of the dynamic balance between squamous and glandular carcinoma components of ASC. These reversely expressed circRNAs may play a key role in the differentiation process of reserve cells into disparate cervical cancer variants.

Potential regulatory roles of circRNAs were further investigated conducting GO and KEGG analysis to annotate BP, CC and MF of host genes. GO results indicated the most significant enriched terms were “metabolic process”, “intracellular”, and “molecular_function” both in SSC and ADC. This observation further supports the reserve cell differentiation theory. In KEGG pathway analysis the most significant enrichment pathway among the three variant groups was “ubiquitin mediated proteolysis”, while the enriched pathway containing the most gene symbols was “Endocytosis”.

In HPV-mediated cervical cancer, the two key oncoproteins E6 and E7 of oncogenic viruses are encoded and they have been shown to have transformation properties. The viral E6 protein physically interacts with p53 through an association mediated by the endogenous E6-AP, ultimately promoting ubiquitination and degradation of p53 in an ATP-dependent manner (Scheffner et al., 1993). It is thought that p53 exerts its growth-suppressive effects through loss of apoptotic function or aberrant checkpoint activity, and its degradation *via* the E6/E6-AP causes carcinogenesis (Vu & Sakamoto, 2000). E7 has a function in cellular transformation through the binding and inactivation of pRB, which interferes with centrosome duplication, ultimately leading to aneuploidy (Howley, 2006). Emerging evidence indicates E7 can inactivate tumor suppressor genes, such as IGFBP-3, in a ubiquitin-dependent way (Santer et al., 2007). Our results derived from circRNAs sequencing, confirmed the “ubiquitin mediated proteolysis” pathway is critical in cervical cancer pathogenesis, regardless of the variant.

We selected eight candidate circRNAs significantly expressed for which the host genes were identified to be part of the “ubiquitin mediated proteolysis” pathway, and we verified their expression by qRT-PCR. We predicted all potential target miRNAs of the candidate circRNAs, and found circRNAs contained multiple target sites for different miRNAs and the targeted miRNAs interacted simultaneously with circRNAs and host genes, as shown in Fig. 10. The existing mechanism of retroregulation to host genes mediated by candidate circRNAs *via* miRNA targeting deserves more research, especially as recent studies have reported various regulatory mechanisms between circRNAs and host genes (Ashwal-Fluss et al., 2014; Li et al., 2015). With regard to target miRNAs that lacked binding sites to host genes, we analyzed significantly enriched KEGG pathways of their targeted mRNA using the DIANA and Novomagic online platform tool. The first ranked result was “TGF-beta signaling pathway ”. The pathway-related protein TGF- *β*1 was considered to be the most effective promoter involved in transforming fibroblast to cancer-associated fibroblasts (CAFs), performing a dominant role in inducing tumor differentiation and progression (Weber et al., 2015).

Four candidate circRNAs displayed reverse abnormal expression between SCC and ADC. This is an interesting hint for exploring cervical cancer variant’s differentiation process from the perspective of specific circRNAs and their relative signaling pathways.

## Conclusions

In conclusion, we investigated circRNA profiles in different cervical cancer variants using RNA-seq analysis. Our findings expand the current knowledge regarding the biology of circRNAs and their regulatory roles in cervical cancer pathogenesis and differentiation. The newly identified network reveals a novel mechanism circRNAs affect host genes. We recommend further studies to fully understand the mechanism underlying these processes.

##  Supplemental Information

10.7717/peerj.14759/supp-1 Supplemental Information 1 Supplemental table and files

## Additional Information and Declarations

### Data Availability

The following information was supplied regarding data availability: The raw sequencing data are available at NCBI’s Gene Expression Omnibus (GEO): GSE208089.
