# Supplementary material for: Comprehensive profile and contrastive analysis of circular RNA expression in cervical squamous carcinoma and adenocarcinoma
Source: PeerJ. 2023 Jan 26;11:e14759. doi: 10.7717/peerj.14759 (PMC9884480; doi:10.7717/peerj.14759)
Supplement: Supplemental Information 1 [file peerj-11-14759-s001.zip › Supp tables and figs 77018/File_S1-S9 legends.docx]

**File S1. CircRNA identification results.**

**File S2. CircRNA coding potential prediction.**

**File S3. Functional enrichment analysis of differentially derived circRNA genes.**

**File S4. List of differentially expressed circRNAs.**

**File S5. The original composition data of Figure 5 and Figure 9.**

**File S6. KEGG pathway analysis on the host genes of reversely expressed circRNAs between SCC and ADC.**

**File S7. KEGG pathway analysis on the host genes of circRNAs from the three intrinsic paired groups.**

**File S8. Target location analysis of the key candidate circRNAs and microRNAs.**

**File S9. KEGG pathway analysis of the target genes of miRNAs irrelevant to ubiquitin mediated proteolysis pathway.**
