# Supplementary material for: Comprehensive profile and contrastive analysis of circular RNA expression in cervical squamous carcinoma and adenocarcinoma
Source: PeerJ. 2023 Jan 26;11:e14759. doi: 10.7717/peerj.14759 (PMC9884480; doi:10.7717/peerj.14759)
Supplement: Supplemental Information 1 [file peerj-11-14759-s001.zip › Supp tables and figs 77018/TableS1.docx]

|  |  |  |
| --- | --- | --- |
| **CircRNAs** | **Forward primer (5′ to 3′)** | **Reverse primer (5′ to 3′)** |
| hsa_circ_0035811 | GGCAGTTGTACACACCATGG | TCCTTCAGCCATTAGTCCTCT |
| hsa_circ_0000989 | AGACCCAAGCTCAGGAGTTG | CCACCTGGTTTAGCTGTCAC |
| hsa_circ_0005325 | AGATAACAGAGCTGCCCGAA | CATCAATAGTATCCAGATCAATCTTT |
| hsa_circ_0004503 | CTACGATCACAGTGGTCTCCA | CGTGCCAGATCATTCAATTCTTT |
| hsa_circ_0005728 | TCCCAACAGATTACCCCTTCA | CGTGCCAGATCATTCAATTCCTT |
| hsa_circ_0018484 | ACATAGGGCAGCACACTTCT | TGTGAAAAGCTTTGATCTCCCC |
| hsa_circ_0004258 | GATGGGGACGCAACAAGTTT | GCAACAACCTGCTCTTGGTT |
| hsa_circ_0059960 | AGCGTATCTTGAACAAACCAGT | GGGGACTGAAGCTCATTATCTG |
| hsa_circ_0070648 | TGTGTCTGATATAGATGTTCCTGA | AAACTGCAGGTTGAGGAGGA |
| hsa_circ_0027966 | GGAGAATTGCCTGATGAACCC | AGAAGGACTGGATCTGTAACACA |
| hsa_circ_0077817 | GGCCCAATGAATATCAGGTTCC | GCAGCTGAAGTCTGGCTTTT |
| hsa_circ_0037710 | GCAGAAGCCTATGAGGAACA | GTGTGGAAGGAGGCAGTACA |
| hsa_circ_0023555 | CCAGCATCAGTGATTTCCTCA | GCCTTGGGATAATTCAGCACTA |
| hsa_circ_0025969 | GGCAGAAGAGACATGAACTGG | AGCCAGTGTCAAAATGTTCGA |
| hsa_circ_0002115 | TGAACACAGGGACCCTTGAA | TTCTCCAACATCACGTCCCT |
| hsa_circ_0073128 | TGACTCAGAAACTACAGGGAACA | CCACTGGCCAAACTTCTGAG |
| hsa_circ_0054226 | GGCGTGTTTCATTGTCTCCA | TGATCTTCCTGGCACTGATGT |
| hsa_circ_0003690 | TATCCCAACTCCCCAGCAAG | CAACAGCTTCCTTTCCCTGC |
| β- actin | ATTCCTATGTGGGCGACGAG | ACGGCAGAAGAGAGAACCAG |
|  |  |  |
