# Supplementary figures and images for: Comprehensive profile and contrastive analysis of circular RNA expression in cervical squamous carcinoma and adenocarcinoma
Source: PeerJ. 2023 Jan 26;11:e14759. doi: 10.7717/peerj.14759 (PMC9884480; doi:10.7717/peerj.14759)

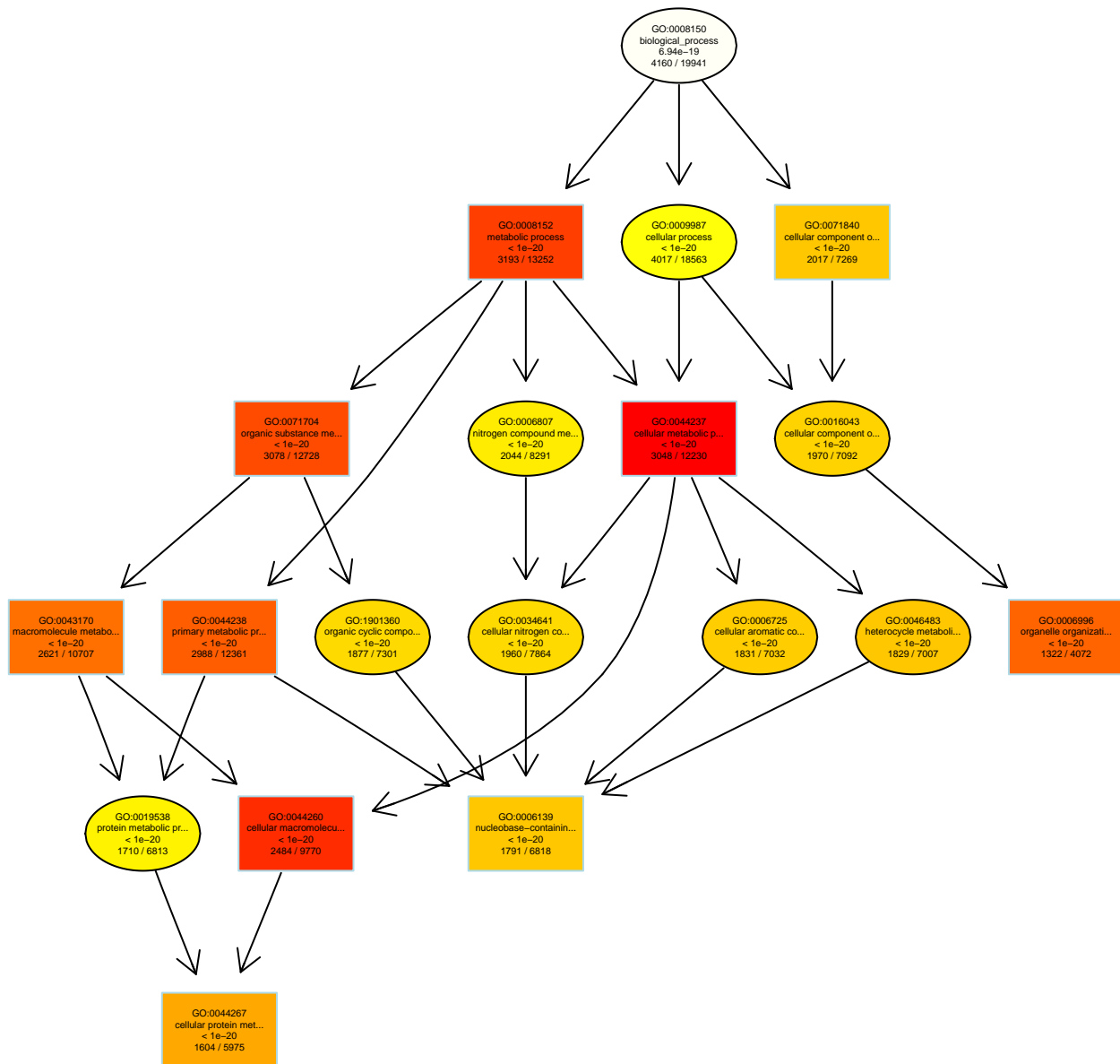

Supplement: Supplemental Information 1 [file peerj-11-14759-s001.zip › Supp tables and figs 77018/File S3/A_1_vs_A_2_Enriched_GO_bp_DAG.pdf]

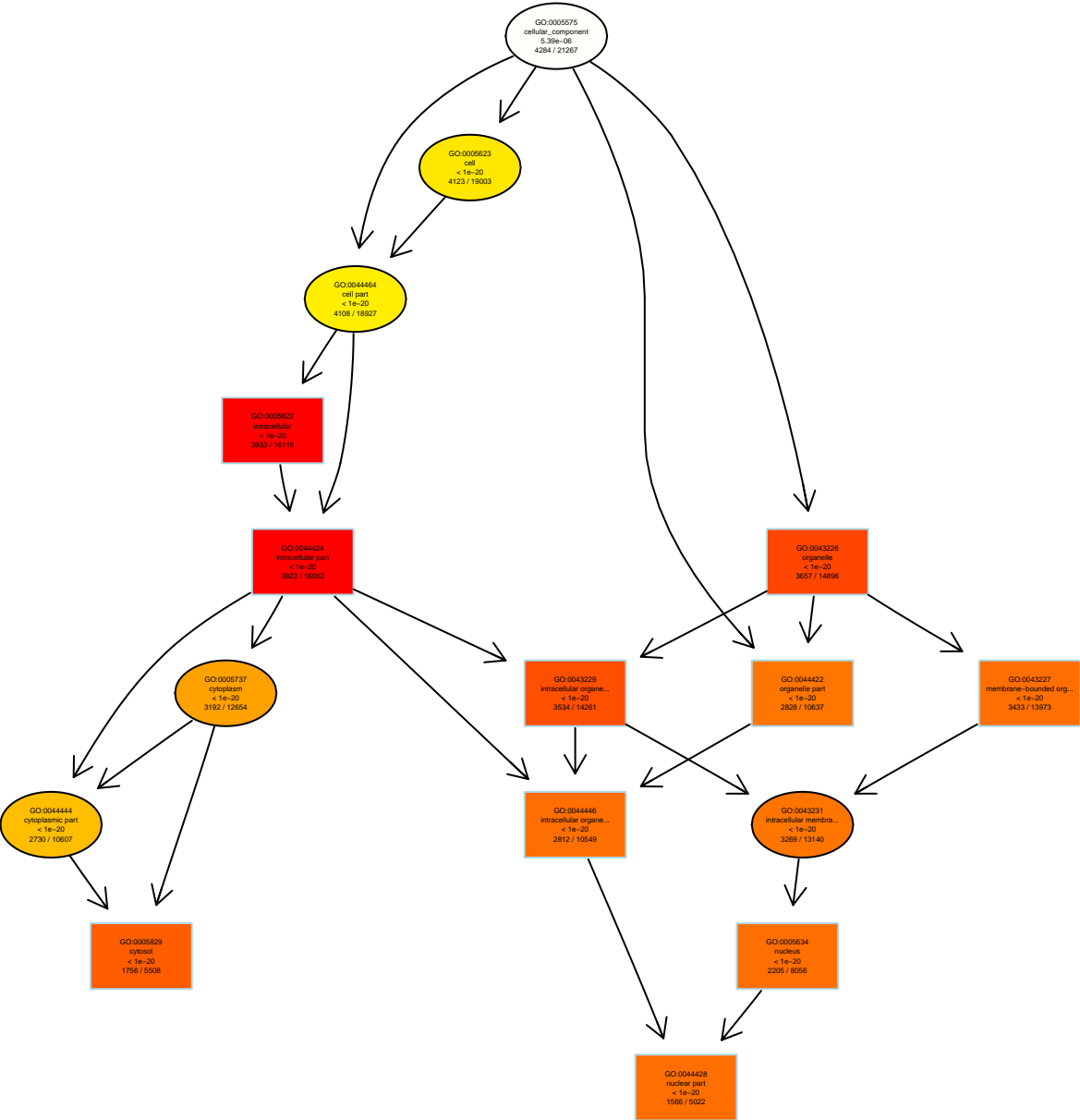

Supplement: Supplemental Information 1 [file peerj-11-14759-s001.zip › Supp tables and figs 77018/File S3/A_1_vs_A_2_Enriched_GO_cc_DAG.pdf]

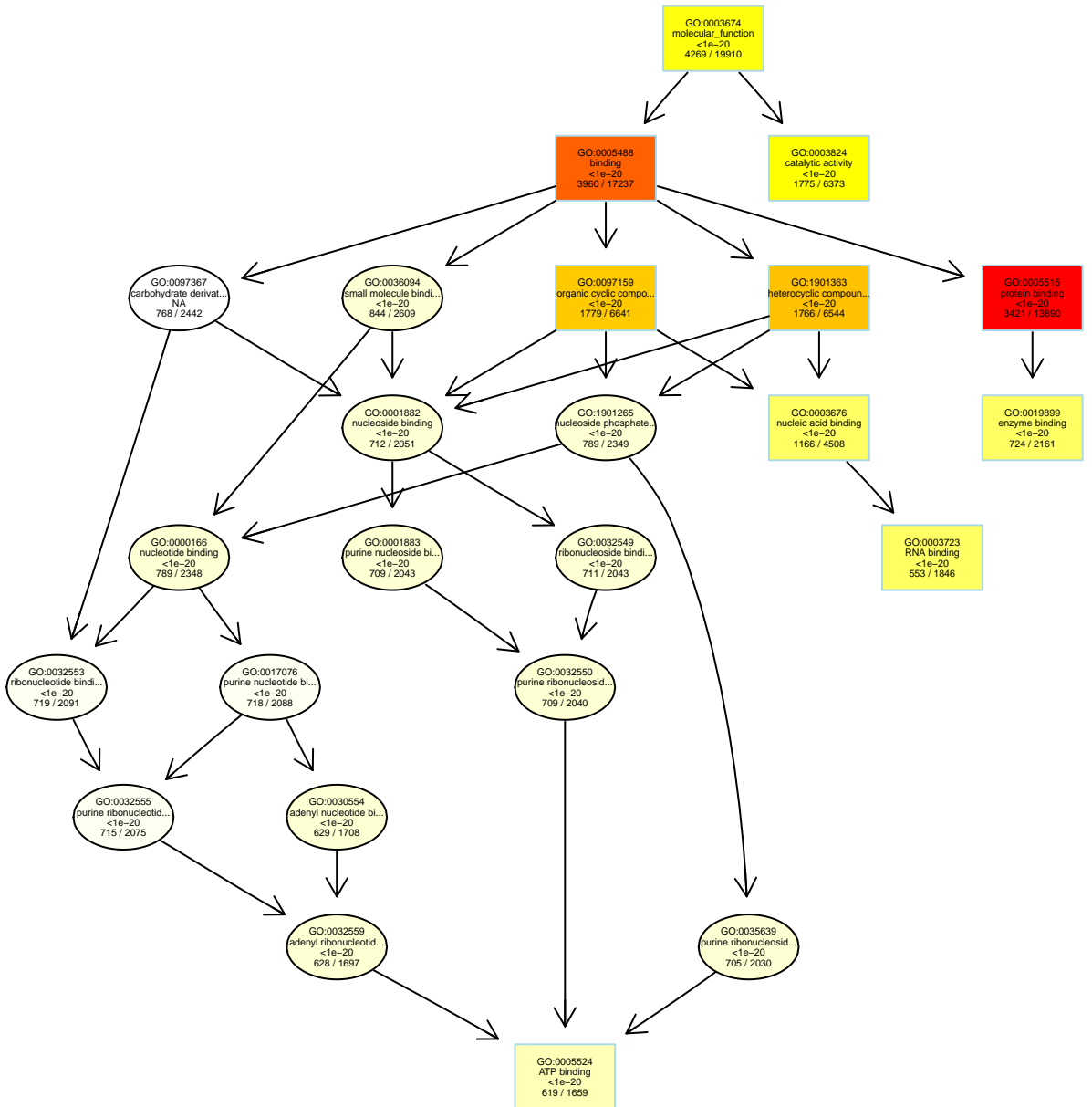

Supplement: Supplemental Information 1 [file peerj-11-14759-s001.zip › Supp tables and figs 77018/File S3/A_1_vs_A_2_Enriched_GO_mf_DAG.pdf]

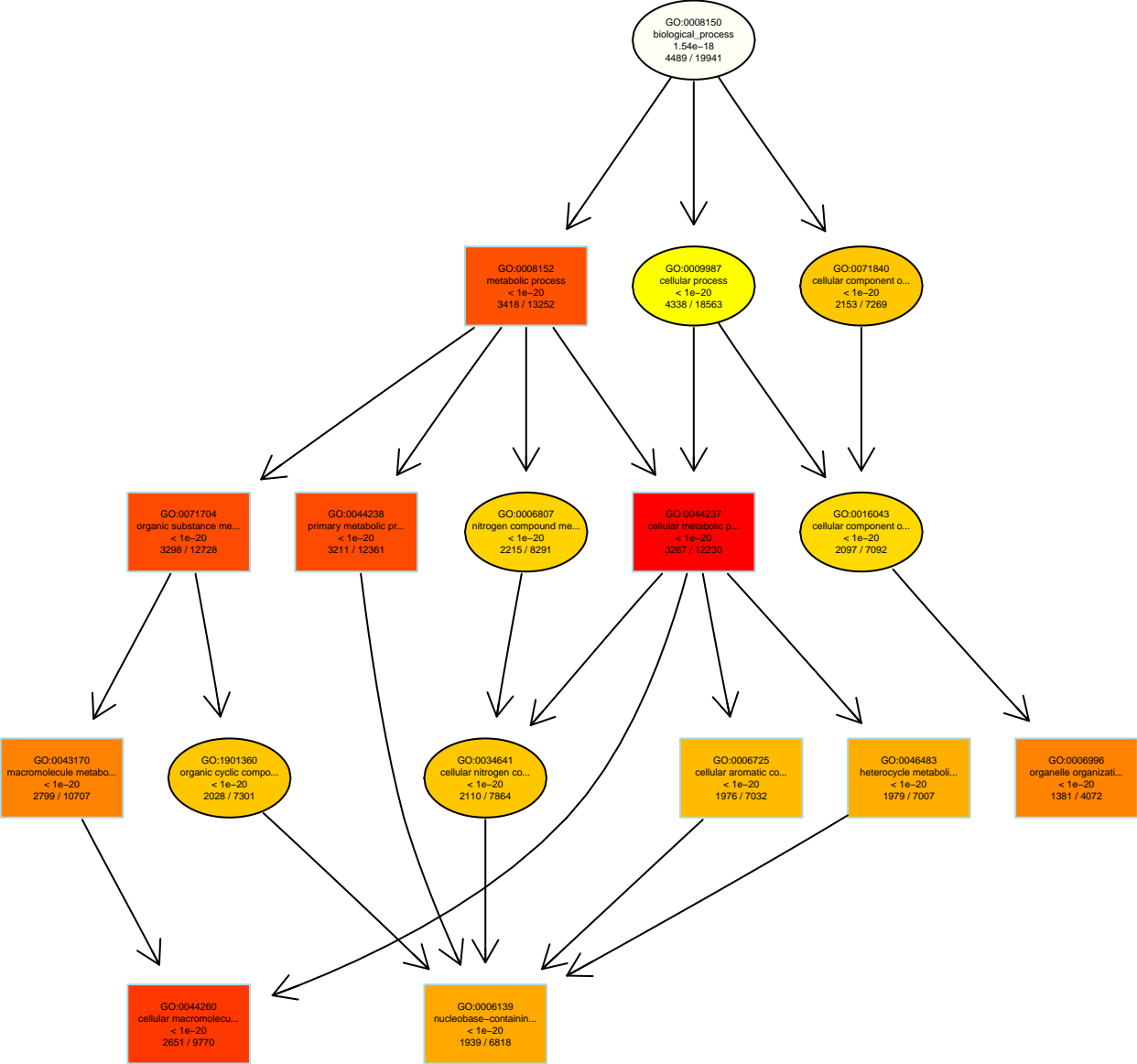

Supplement: Supplemental Information 1 [file peerj-11-14759-s001.zip › Supp tables and figs 77018/File S3/AS_1_vs_AS_2_Enriched_GO_bp_DAG.pdf]

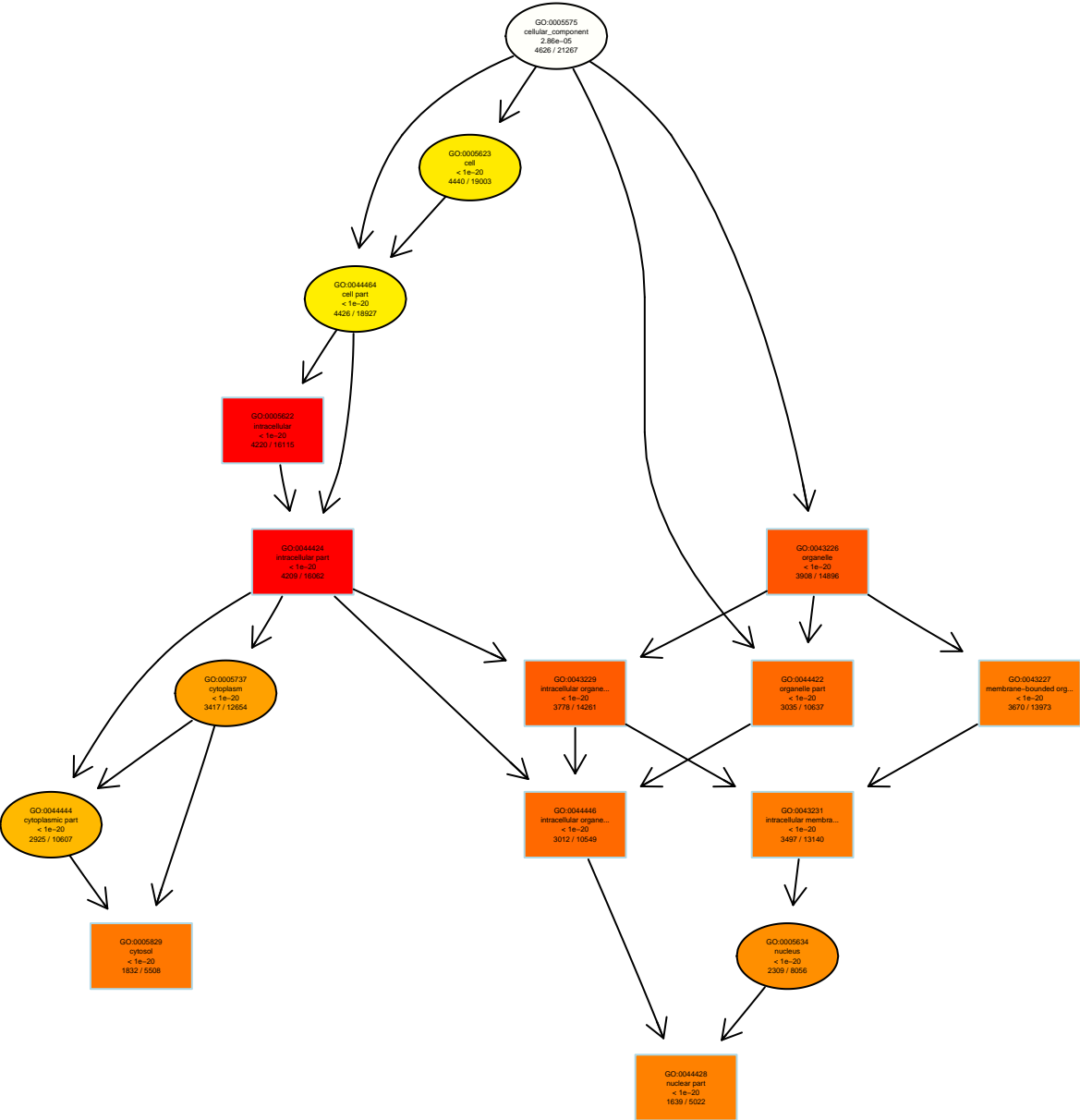

Supplement: Supplemental Information 1 [file peerj-11-14759-s001.zip › Supp tables and figs 77018/File S3/AS_1_vs_AS_2_Enriched_GO_cc_DAG.pdf]

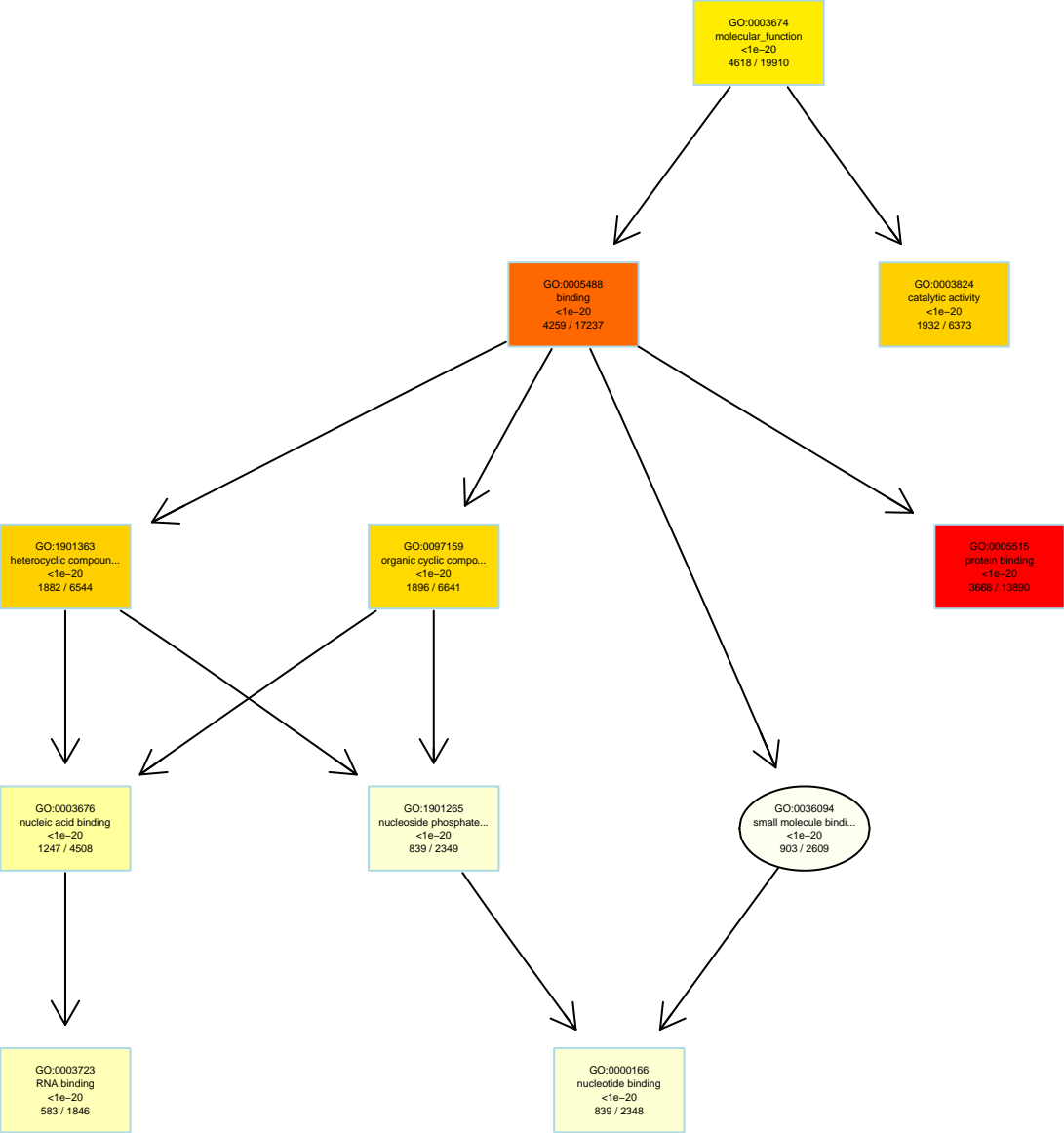

Supplement: Supplemental Information 1 [file peerj-11-14759-s001.zip › Supp tables and figs 77018/File S3/AS_1_vs_AS_2_Enriched_GO_mf_DAG.pdf]

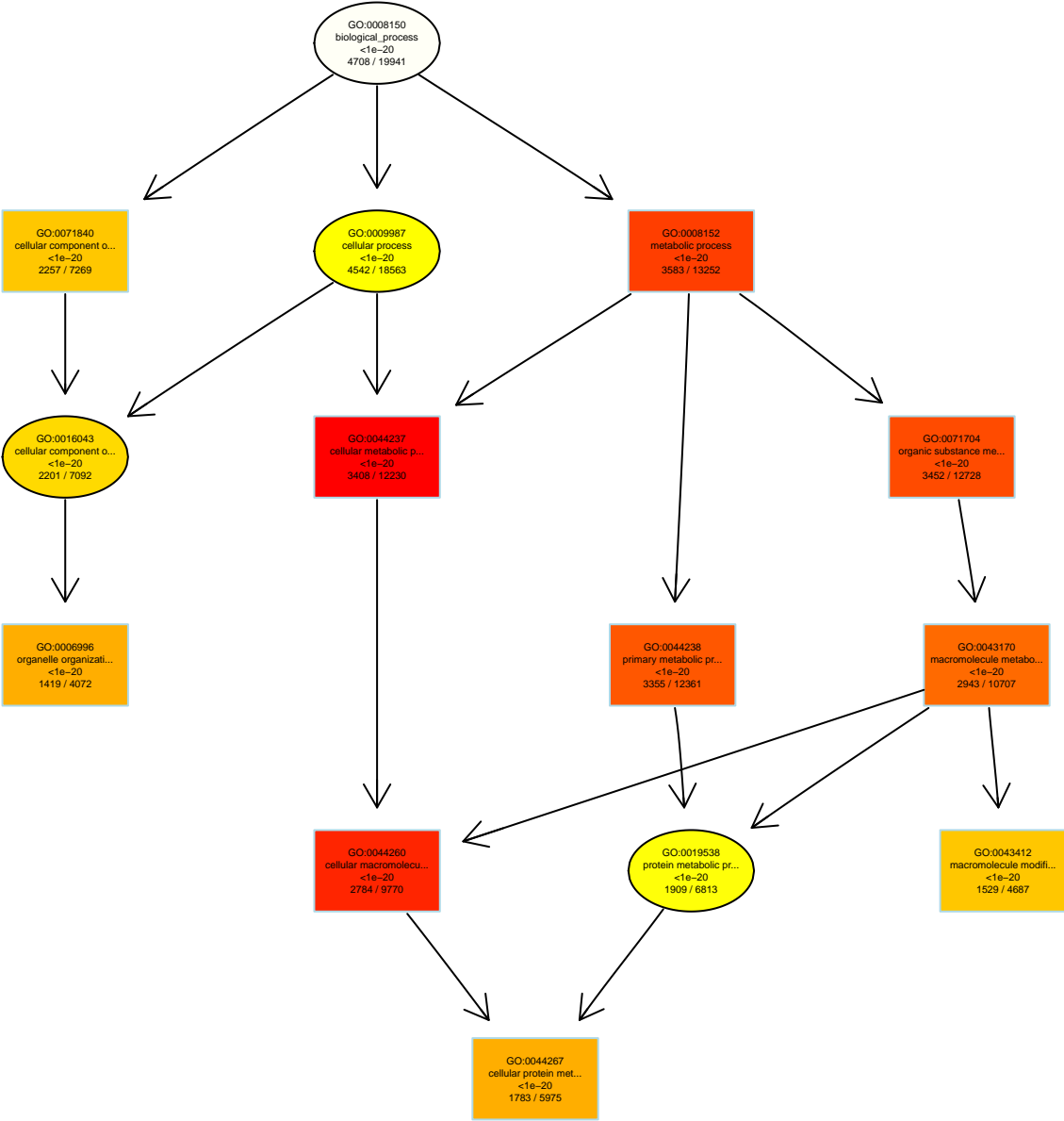

Supplement: Supplemental Information 1 [file peerj-11-14759-s001.zip › Supp tables and figs 77018/File S3/S_1_vs_S_2_Enriched_GO_bp_DAG.pdf]

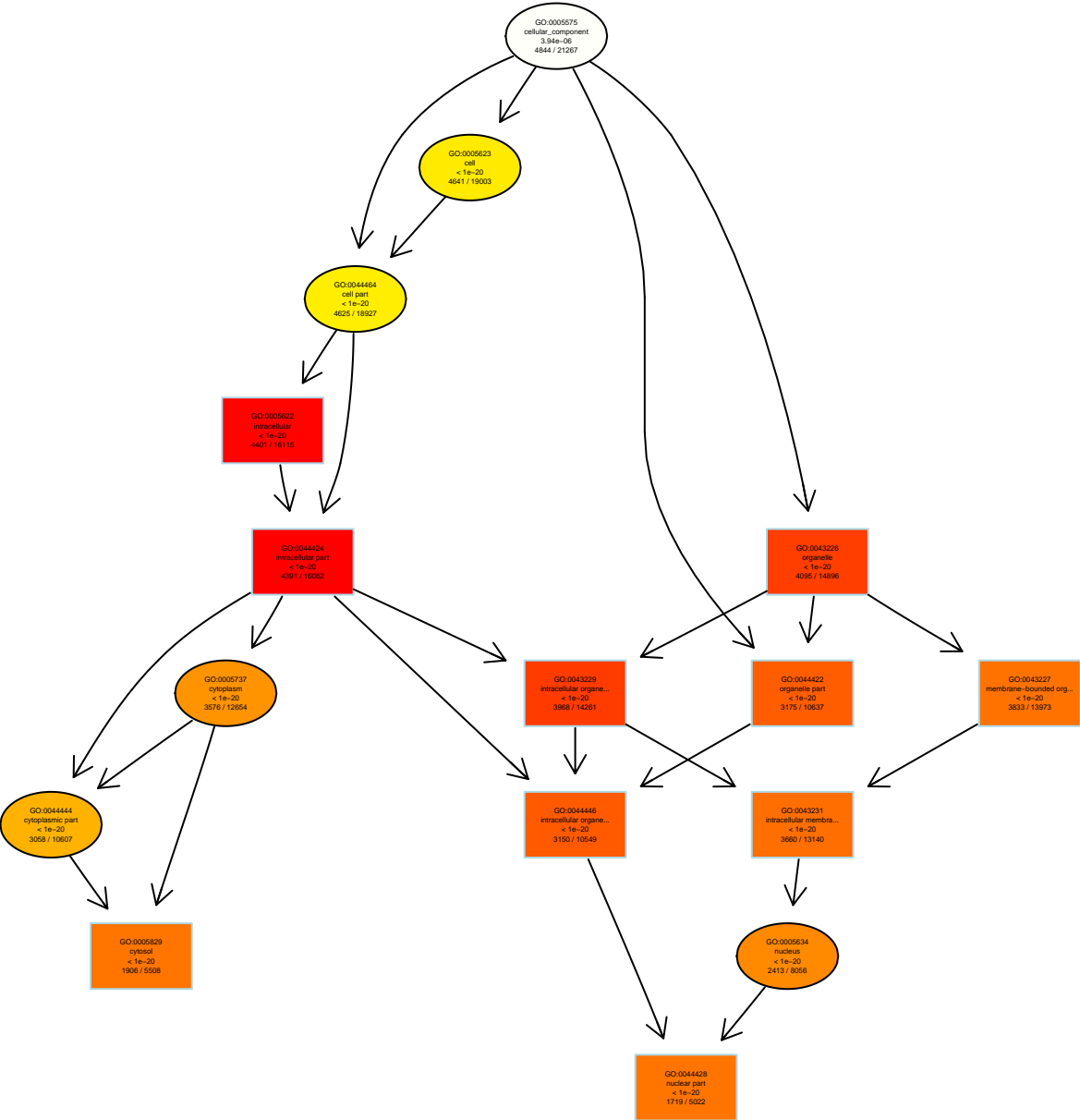

Supplement: Supplemental Information 1 [file peerj-11-14759-s001.zip › Supp tables and figs 77018/File S3/S_1_vs_S_2_Enriched_GO_cc_DAG.pdf]

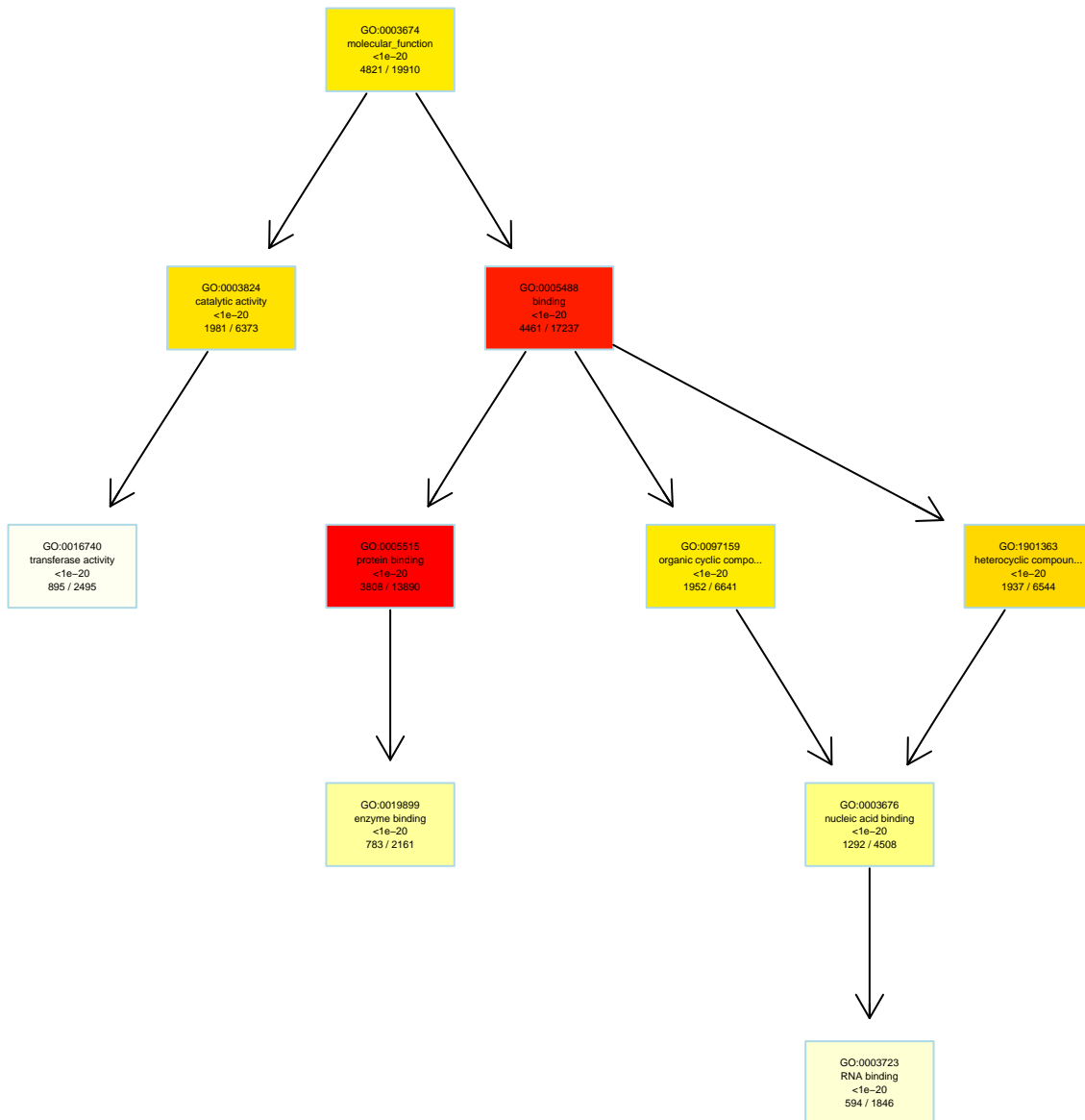

Supplement: Supplemental Information 1 [file peerj-11-14759-s001.zip › Supp tables and figs 77018/File S3/S_1_vs_S_2_Enriched_GO_mf_DAG.pdf]
